# Gel Carriers for Plant Extracts and Synthetic Pesticides in Rodent and Arthropod Pest Control: An Overview

**DOI:** 10.3390/gels8080522

**Published:** 2022-08-20

**Authors:** Jawad Ali Shah, Tomas Vendl, Radek Aulicky, Marcela Frankova, Vaclav Stejskal

**Affiliations:** 1 Department of Plant Protection, Faculty of Agrobiology, Food and Natural Resources, Czech University of Life Sciences Prague, 16500 Prague, Czech Republic; 2 Crop Research Institute, Drnovska 507/73, 16106 Prague, Czech Republic

**Keywords:** hydrogels, polymers, nanotechnology, insecticides, rodenticides, formulations, essential oils, plant extracts, integrated pest management, vector control

## Abstract

Insecticides and rodenticides form the basis of integrated pest management systems worldwide. As pest resistance continues to increase and entire groups of chemical active ingredients are restricted or banned, manufacturers are looking for new options for more effective formulations and safer application methods for the remaining pesticide ingredients. In addition to new technological adaptations of mainstream formulations in the form of sprays, fumigants, and dusts, the use of gel formulations is becoming increasingly explored and employed. This article summarizes information on the current and potential use of gel (including hydrogel) and paste formulations against harmful arthropods or rodents in specific branches of pest management in the agricultural, food, stored product, structural wood, urban, medical, and public health areas. Due to the worldwide high interest in natural substances, part of the review was devoted to the use of gels for the formulation of pesticide substances of botanical origin, such as essential or edible oils. Gels as emerging formulation of so called “smart insecticides” based on molecular iRNA disruptors are discussed.

## 1. Introduction

Soft gels as material consisting of two or more components, one of which is a liquid, found applications in wide range of products such as foods, agrochemicals, pharmaceuticals, cosmetics, soft-optical devices, art conservation preparations, paints, adhesives, etc. [1]. Gels were also proposed as basis of technologies for the removal of environmental pollutants [2] or of sensors employed in various environmental and biological applications [3]. Various soft gels and pastes have also been used for some types of traditional insecticide [4,5] and rodenticide formulations [6]. In recent decades, especially with the advent of advances in encapsulation and nanotechnologies [4,7,8,9], researchers focused their attention on pesticide gel application to new areas of protection of human resources, health, and environment against harmful and invasive organisms. Pesticide gels are thus beginning to be a complement, or in some cases an important environmentally safer alternative, to traditional pesticide formulations.

Traditional types of pesticide preparations include solid, gas, liquid, and gel formulations [10]. Each pesticide’s active ingredient must be suitably formulated using a proper carrier, synergists, additives, and other formulation components. Optimized commercial formulations not only prolong the residual effect and increase the biological efficacy of pesticides on pests but also increase their safety to humans, non-target organisms, and the natural environment. Since pest resistance to pesticides continues to increase and entire groups of chemical active ingredients are restricted or banned, manufacturers are looking for new options for more effective formulations and safer application methods for the remaining pesticide ingredients. Thus, advanced application techniques [11] and formulations of insecticides and rodenticides based on polymers, nanotechnology, and encapsulation of active ingredients are gradually coming to the forefront of pesticide manufacturers’ concerns [12,13,14,15,16,17,18]. According to Tay et al. [4], the most frequently used polymeric encapsulated wall materials include gelatin, gum arabic, starch, sugar, ethyl cellulose, carboxymethyl cellulose, paraffin, polyvinyl alcohol, polyethylene, polypropylene, polystyrene, polyacrylamide, polyethers, polyesters, polyamides, polyureas, polybutadiene, polyisoprene, polysiloxanes, polyurethanes, epoxy resins, and inorganic silicates. Among other perspective preparations, gels and hydrogels are formulations that are becoming increasingly scientifically explored and employed in practice during the past few decades. To our knowledge, there is no general inventory summarizing the usage of pesticide gels for various areas and branches of pest control. Since insecticide aerogels were extensively covered by many studies and reviews [19], they are not included in the study. This review article summarizes information on gel, hydrogels in particular, and paste formulations against harmful arthropods and rodents. This is not intended to be a systematic review of the field or of the chemical characterization of pesticide gels. Instead, it aims to generally summarize the main areas of current use of gels against harmful organisms and to present potential future applications in various areas of pest control.

## 2. Overview of Pest Control Areas Regarding Usage Gel Formulations

Protective chemicals designed and registered to control harmful organisms are generally called pesticides. In European Union (EU) legislation, pesticides are divided into biocides and plant protection products. The active ingredients of pesticides include a large, diverse set of chemical groups and can be either natural or synthetic in origin [10,20]. Currently, the most extensive research is conducted on the substances and their carriers (oils, algae gels), which are of natural origin and are thus often easily biodegradable. For example, edible or essential oils [11,21] are used as natural pesticides and repellents. In terms of target animal pests, the most important categories of pesticides are insecticides (including acaricides), which are designed to control harmful arthropods (i.e., insects and mites), and rodenticides, which are designed to control rodents or other harmful vertebrates. Insecticides and rodenticides are used globally in various spheres of human society. They are widely used along the food production chain, from primary agricultural production to commodity storage and food production. Pesticides are an important element of national and international phytoquarantine and environmental protection against invasive organisms. Another important area of their use is the control of pests of medical importance. Here, pesticides are an important tool to ensure public health.

**Figure 1:**
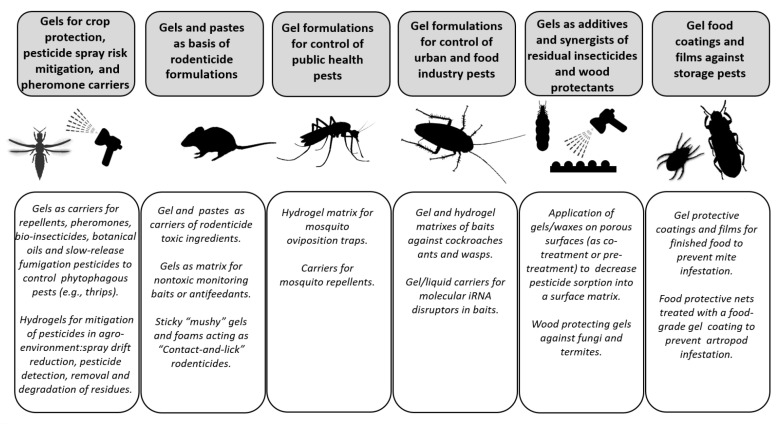
Graphical illustration of usage of gels—including hydrogels—in various areas of pest control and mitigation of pesticide environmental impacts.

Based on the openly published literature records, this review recognized main areas for which pesticide gels were suggested. Their inventory is summarized in Figure 1, and the structure of the review is arranged accordingly. Due to the worldwide high interest in natural substances, a separate chapter is devoted to the use of gels for the formulation of pesticide substances of botanical origin, such as essential or edible oils. The review also recognized gels as emerging formulations—so-called “smart insecticides”—based on molecular iRNA disruptors.

## 3. Gels in Plant Protection and Pesticide Sprays Risk Mitigation

Gel-based formulations, and in particular highly sorptive hydrogels, are being increasingly explored to ensure environment safety and sustainable agricultural production. Information on the research or application of gels and hydrogels in practice can be found in several dedicated reviews on this topic [22,23,24,25,26]. According to the reviews, gels and hydrogels have much broader applications in agriculture than only plant protection and pest control. However, regarding topics related to plant protection, two broad areas of research and application of gels have been identified during preparation of this review. The first area was the role of hydrogels as carriers of direct pest control agents and as indirect mediators which increase plant health. The second area was the mitigation of pesticide negative side effects, such as water or soil contamination by pesticides residues.

### 3.1. Gels as Carriers for Bioinsecticides and Slow-Release Pesticides or Pheromones in Crop Protection

Gel preparations are important as means of both direct and indirect plant protection. The indirect protection is based on providing a controlled supply of moisture and/or nutrients. Additionally, gels serve as a supportive means of improving overall plant/seed resilience and health [27,28]. Healthy and unstressed plants and seeds are more tolerant/resistant to pest infestation or disease infection. Hydrogel-based products are considered soil conditioners and yield enhancers because of their ability to increase the water holding capacity of the soil substrate and improve soil texture [29]. They do not only increase soil substratum water capacity and improve the soil structure but are also capable of releasing the retained water and nutrients over an extended period of time [30]. Regarding the use of gels and hydrogels as supporting forms of irrigation to improve the physiological state of plants, critical papers showing both the general limitations of their biological activity and the specific inhibitory effect of different soil conditions can be found [31,32].

As direct means of protection, gels can serve as carriers of pesticides and biocontrol agents within the framework of the so-called integrated pest management (IPM) system. Gels, as carriers of direct control agents, have unique properties, especially in terms of the so-called controlled long-term release of active pesticide substances [24,33,34,35,36] or pheromones [37]. For example, controlled release of chlorpyrifos [6] and carbaryl beads [5] using hydrogels was described. Importantly, the authors claimed that the PA-CA hydrogel possessed biocompatibility with *Escherichia coli*, thereby also positively addressing a biosafety issue [38]. In terms of various higher taxa groups of target pest organisms, highly sorptive pesticide hydrogels have been formulated not only as insecticides but also as fungicides [39,40,41] and herbicides [42,43,44,45]. Apart from pesticide synthetic compounds, hydrogels were suggested as a formulation matrix for natural compounds [46] and bioagents [47]. The natural-based formulations may be potentially employed in situations in which biocontrol is required, such as in some types of organic farming systems. Ropek and Kulikowski [47] elaborated a hydrogel-based biopreparation containing entomopathogenic fungus *Beauveria bassiana*. Although some authors state that high temperatures have rather negative effects on the efficacy of hydrogel-based insecticides [4], Ropek and Kulikowski [47] reported that the *B. bassiana* gel preparation acted faster at temperatures of 25 and 30 °C than at low temperatures.

Although interesting hydrogel application systems have been proposed for the control of agricultural pests, the application of many of them remains at the research or finished technical solution stage. One of the frequent reasons for the difficulty in transferring research results into practice is the prohibitively high cost of international pesticide registrations.

### 3.2. Hydrogels for Mitigation of Pesticides in Agroenvironment (Spray Drift Reduction, Pesticide Detection, Removal and Degradation of Residues)

Gels were suggested not only for pesticide efficacy enhancement and prolonged degradation time of pesticide residues after their application in field conditions, but also for mitigation of pesticide negative effects associated with plant protection [48]. Hydrogels were designed as pesticide application formulations to reduce insecticide spray drift during spraying under field conditions. Song et al. [49] explored possibilities of reduction of pesticide spray drift using folate/Zn^2+^ supramolecular hydrogels. These authors designed novel organic solvent-free hydrogel showing biocompatibility and biodegradability. The primary method by which the hydrogels reduced drift was by increasing droplet size, which was caused by the three-dimensional network of the internal structure of the gel. In our view, such a line of research and development points in the direction of new strategies for spray drift reduction in terms of pesticide formulation. Thus, it expands the possibilities of using supramolecular hydrogels in agriculture. In addition, it has been revealed that preparation based on hydrogels can contribute to the targeted degradation of pesticides in contaminated soil. For example, Yang et al. [50] found that superabsorbent hydrogel coating increased degradation and decreased the formation bound residues, such as carbendazim, in soil. Several procedures were explored in terms of hydrogel capacity for removal of various pesticides from the contaminated water. The incentive for such studies was to develop practical procedures and formulations of hydrogels as preparations for the decontamination of water from insecticides. The results achieved by Aouada et al. [51] suggested that PAAm-MC hydrogels are potentially viable absorbents for removal of paraquat pesticide from aqueous solution and cleaning water contaminated with dyes, heavy metals, and other pesticides. Alammar et al. [52] constructed neonicotinoid-scavenging nanocomposite hydrogels. Therefore, Gosset et al. [53] developed sensors based on encapsulated algae for pesticide detection in water. Recently, a new concept of a smart MOF-on-MOF hydrogel serving for visual detection of pesticide residues to enable effective removal of pesticides from contaminated environment was proposed [54].

## 4. Rodenticide Gels and Pastes

Rodents are serious agricultural, storage, food industry, public health, and veterinary pests. They are highly damaging to agricultural crops [55] and contaminate environment with feces containing pathogens [56,57]. Designing the appropriate bait formulation for the target rodent species is difficult in terms of attractiveness, palatability, and safety. Active ingredients of rodenticides are acute or chronic substances [56]. Chronic anticoagulants accumulate in the body of poisoned rodents [58,59], and thus, secondary poisoning may occur after consumption by predators. Therefore, effective and safe rodent control and monitoring is a challenge. New works on innovative rodenticide formulations associated with gels (that are often called rodenticide “soft baits”), polymers, and encapsulated active compounds [22] point the way towards further development to effectively manage rodents and mitigate the negative effects of rodenticides [14].

### 4.1. “Contact-and-Lick” Rodenticide Sticky Gels and Foams

The concept of using contact rodenticides, which involves the rodent soiling itself with a contact toxic substance, was developed historically long ago. In the 1950s, several historical formulations of greasy and sticky poisonous pastes, saps, and greasy sticky material (called “mushy mass”) were developed and used for rodent crop pests, especially common voles (*Microtus arvalis*) [60] in Europe. Due to high labor demands, agricultural contact adhesive rodenticides are rarely used these days. However, the concept of soft adhesive toxic coat contaminants (foam with anticoagulants) has recently been refreshed for the control of synanthropic rodents, such as Norway rats (*Rattus norvegicus*), roof rats (*R. rattus*), and house mice (*Mus musculus*). A contact rodenticide formulation in the form of a foam (Racumin Foam—Bayer, Germany) with coumatetralyl as the active ingredient is currently registered in a number of EU countries and elsewhere. Although this product is used in practice, no freely available peer-reviewed scientific study on its efficacy has been published to our knowledge.

### 4.2. Toxic Bait Gels and Pastes (Soft Baits)

Wilson [61] described a brief historical development of bait formulations in his paper titled “Evolution of rodenticides”. In the historical timeline, the “oldest” solid baits were based on poisoned grain. The latest rodenticide generation of bait formulations was identified by Wilson [61] as soft baits, which are based on gels and pastes. Cornwell [22] was one of the first who extensively tested encapsulated forms of various rodenticide active ingredients formulated with ethyl cellulose, gelatin, gelatin/gum arabic, gelatin/carrageen, polyester wax, and polawax. Currently, soft baits for rodents are commonly produced and used. Pastes and gels are applied to target sites using various handheld injection press devices or are preprepared by the manufacturer in small packages for direct application; the latter formulations are known as ready-to-use rodenticide sachets. There are a number of historical and recent scientific studies that demonstrate the attractiveness, palatability, and efficacy of different formulations of soft baits on house mice, roof rats, and Norway rats under different environmental conditions [62,63,64,65]. Soft baits have been even evaluated for controlling rodents outside buildings and for environmental protection against invasive pests (e.g., the Polynesian rat, *R. exulans*) [66].

### 4.3. Nontoxic Monitoring Gel Baits and Antifeedants

Gel soft baits can be used not only for direct rodent control but also as a suitable matrix for the formulation of repellents and antifeedants or as attractants for rodent monitoring using nontoxic baits. Rodent repellents and antifeedants, such as alginate-based microcapsules containing eucalyptus oil, are still mostly in the research stage [67]. Nevertheless, nontoxic soft baits for rodent monitoring are already being produced and used in practice. Their advantage is that they do not need to be registered as pesticides in most states. There have been published results of lab and field tests on different formulations of commercial soft nontoxic baits for monitoring synanthropic house mice and roof rats in indoor environments and agricultural orchards [65,68,69,70].

## 5. Gels for Risk Mitigation of Mosquitoes

Mosquitoes are among the most important vectors of pathogens which are the causative agents of serious diseases such as malaria [71]. Mosquito-risk-reduction strategies include, among others, either methods that prevent mosquitoes from coming into contact with humans or methods that reduce the size of mosquito populations. In particular, repellents serve as a personal protective shield (applied to skin, clothing, or netting over beds) against mosquito contacts with humans. There are a number of detailed published reviews regarding insecticidal repellents [72,73] covering different formulations, such as lotions, sprays, gels, creams [74], and polymers [75]. Therefore, in this much more general review, we limited ourselves to providing a few examples regarding the development of hydrogel mosquito repellent formulations. For example, Milutinović et al. [76] demonstrated that the hydrogel formulation based on polyacrylic acid containing 5% DEET (*w*/*w*) could serve as a suitable vehicle for repellent preparations containing DEET, and Pinto et al. [77] characterized a new repellent formulation based on nanostructured hydrogels. Delong et al. [78] screened various natural extracts in order to develop a new hydrogel formulation (pHEMA hydrogels with pendant triazinyl-β-cyclodextrin) containing repellents of plant origin, which may provide a suitable, eco-friendly approach to achieving mosquito bite protection. They found that methyl salicylate possessed an optimal stability, and thus, it achieved a longer duration of protection with higher repellent activity. Kumar et al. [79] showed the promising repellent activity and delayed release of citronella-oil-microsponge-loaded hydrogel. Most recently, Rogeiro et al. [80] developed nanoparticle mosquito repellent based on a slow-release system composed of zein nanoparticles containing the encapsulated active agents icaridin and geraniol incorporated in a cellulose gel matrix.

Elimination of mosquito breeding sites, application of pesticides and bioagents, egg and adult trapping, and other measures are among the methods of direct control of mosquito population density. As important tools for mosquito-borne malaria control in the outdoor environment, both synthetic and natural substances are finding application, whether as repellents or as population density reduction agents [81,82,83]. Recently, Mapossa et al. [84] reviewed new various controlled-release formulations for mosquito control, such as polymer microcapsules, polymer microporous formulations, polymer micelles, nanoemulsions, solid lipid nanoparticles, liposomes, and cyclodextrins. In addition to traditional gels, new hydrogels are among the formulations that already have or are finding new applications for mosquito control. In addition to attractant or repellent formulations, they may be employed as carriers of larvicide formulations [85] or in mosquito ovitraps [86,87,88]. Friuli et al. [88] validated a new recipe of hydrogels to imitate the natural oviposition site’s conditions in order to incorporate them inside “lure-and-kill” ovitraps as a biomimetic oviposition substrate. The study compared oviposition between standard substrates (absorbing paper/masonite) and a hydrogel composition panel under labor field conditions. The tests showed that a 2-hydroxyethylcellulose (HEC)-based physical hydrogel was five times more attractive than the control in a laboratory oviposition assay. Under field conditions, the same hydrogel substrate was more efficient than a standard masonite ovitrap, with a four-times-longer activity duration.

## 6. Gels Baits for Control of Ants, Cockroaches and Other Urban Pests

Insecticide gel baits have the potential to greatly reduce the amount of spray insecticides needed for pest control. Ready-to-use forms of gel and paste baits are delivered to the destination using pressurized propellant-based containers, gun-type applicators with removable injection tips, or by baits in tamper-resistant box stations [89]. Compared to residual spray formulations, baits are relatively less toxic, odorless, and may be applied in minute amounts to areas where residual spray is not permissible [90]. However, it should be noted that the bait efficacy is tightly dependent on pest movement activity, which is highly influenced by temperature. Thus, a lower temperature threshold for insect movement imposes a limit on bait efficacy [91]. The current baits mainly contain relatively nonrepellent, slow-acting compounds [90,92,93,94]. Most recently, the development of bait technologies for liquid baits with liposomes as carriers of dsRNA has been suggested by several research teams [95,96]. In recent decades, a new generation of attractive (synthetic or natural) baits or gel-based baits, frequently hydrophilic, has been established. Microencapsulated oil bait was constructed for ant control [97]. Novel usage of various types of hydrogels (superabsorbent polymer–SAP [98]) was explored as bait matrix for the risk management of various urban pests [4]. Regarding taxonomical pest spectra, the hydrogel baits were evaluated for control of ants [99,100,101], wasps [4,102], and cockroaches [98]. Hydrogel-based baits have been most extensively evaluated as baits for control of a serious medical and urban pest, the Argentine ant, *Linepithema humile* [101,103,104]. Oladipupo et al. [98] stressed that hydrogels, used as a bait-delivery option, require a relatively small amount of insecticide active ingredient. Hydrogels are capable of absorbing water from a moist substrate, which compensates for water loss through surface evaporation. Hydrogels can be rehydrated via irrigation or rainfall, and the rehydration process allows the hydrogels to attract urban pests again [4]. The tested gel groups included synthetic polyacrylate hydrogels [99] or biodegradable hydrogels of natural origin (alginate hydrogels) [4,100,105]. For example, Tay et al. [100] developed an alginate hydrogel to deliver aqueous bait for pest ant management. The storage of natural and synthetic hydrogel baits hydrated with sucrose solution may be complicated by the fact that they must be stored in a refrigerator to prevent fermentation [4]. Further and more detailed information on various aspects of hydrogel usage and technological development can be found in an extensive review by Tay et al. [4].

## 7. Gels as Additives of Residual Insecticides and Wood Protectants

Food industry facilities and empty grain stores are treated with insecticidal surface residual sprays [10]. The activity and persistence of insecticide deposits largely depend on the particular composition, texture, and porosity of the treated surface. Steel, ceramic, or painted surfaces do not absorb liquids, whereas wood and brickwork have high sorption capacities for many liquid insecticide formulations. To avoid sorption into a porous surface matrix, cotreatment or pretreatment of a surface by means of protective coatings has been developed in the past. For example, Hewlett [106] experimentally documented that pre-treatment of cement with various gelatins greatly prolonged the toxic lifetime of insecticide films. Parkin and Hewlet [107] found that coating bricks with starch paste and water glass increased the activity of DDT and pyrethrins against some species of storage Coleoptera. Some petroleum oil films were highly toxic to tested beetle species (*Sitophilus* sp.) when applied to cement pretreated with gelatin [108]. Tyler and Rowlands [109] mixed carboxymethyl as a protective cotreatment with malathion sprays; it resulted in markedly improved persistence of the residual film on an alkaline cement substrate. Gudrups et al. [110] found that pyrethroid insecticide (permethrin) applied to concrete with a wax polish coating provided control of some storage Coleoptera species for 14 days. The idea of gel co/pretreatment may be an inspiration for technological improvements of plant-based insecticide formulations regarding their field efficacy.

The surface of structural/construction wood (timber) is required to be efficiently treated or impregnated by protective pesticides with long-term stability and activity. In this regards, Obounou-Akong et al. [111] tested hydrogels that fill the tracheid cell walls and lumens, limiting the leachability of boron salts when the wood is humidified. According to these authors, hydrogels appear to be valuable additives for improving boron fixation in wood and developing waterborne wood preservation formulations against harmful organisms, such as fungi and termites.

## 8. Gel Coatings for Finished Food Protection against Mites

In addition to transport purposes, the packaging of agricultural commodities and prepared foods serves mainly to protect them from contamination [112,113] and other negative environmental influences. Packaging is an important element of protection against pests. Various types of packages differ in the resilience to penetration or invasion by various pest species [114,115,116,117]. Vacuum packaging or packaging filled with inert gases can then provide increased food protection [118,119,120]. The real challenge for effective pest protection is unpackaged food. Particularly problematic are those types of food (dried fish, ham, cheese, salami and uncooked, cured, dried, and smoked or unsmoked meat products) that are stored for long periods of time or have to be technologically ripened in conditions that allow them to be attacked by pests. To control these pests, various formulations of nonresidual fumigants and modified atmospheres or residual liquid pesticides dips or films were tested and/or used in practice [121]. Since residues of most pesticides—including acaricides [122]—are not legally acceptable or refused by customers, as alternatives, food-grade protective gel coatings (films) or nets with gels possessing acaricide properties have been proposed [123,124,125,126]. Any type of food coating must not affect the sensory properties of dry-cured hams but should allow water permeability [127]. This limits the number of potential types of chemical gels that can be used for these purposes. One of the potential candidate compounds seems to be propylene glycol. This type of gel is completely miscible with water and many organic solvents and is used in cosmetic and pharmaceutical formulations. Laboratory test by Zhao et al. [125] revealed that food matrix coated with xanthan gum + 20% propylene glycol and carrageenan/propylene glycol alginate + 10% propylene glycol effectively controls mite populations. The researchers also tested various combinations of mechanical packaging in the form of nets with gel-based chemical protectants. For example, Campbell et al. [127] showed that ham nets treated with a food-grade coating of 1% propylene glycol alginate + 1% carrageenan + 40% propylene glycol delivered protective effect not only against acaroid pests but also against some mold/fungi species.

## 9. Gel Formulations with Natural Plant Extracts as Pesticides and Repellents

Recently, plant extracts (e.g., essential and edible oils) have gained popularity. Their use is offered in many areas of pest control. Edible and essential oils have been tested as botanical insecticides against a wide range of pests that attack both stored commodities and standing crops [128,129]. From an ecological and partly toxicological point of view, plant oils are valued because they are biodegradable and thus do not tend to accumulate in the environmental chains. However, this welcome chemical property is associated with the problem of their short bioactivity after application [130]. For that reason, it is important to develop formulations that ensure greater stability and controlled release of the natural substances. Mechanisms of improving essential oil stability include various encapsulation techniques of natural substances by formulating them as nano- and microemulsions [131], microspheres [132], and nanoparticles [133]. The use of highly sorptive hydrogels may also provide higher and longer activity of essential oils applied as pesticides and repellents [46,98,134].

### 9.1. Hydrogels as Matrix for Essential Oils Based Baits or Fumigation Pesticides

Essential oils (EOs) formulated in hydrogels may be used as natural-based pesticides (acting as contact pesticides, fumigants, or baits) for the direct control of pests. For example, Gharbi and Tay [46] conducted a series of fumigation assays to assess the vulnerability of two species of pest thrips (*Frankliniella occidentalis* and *F. insularis*) to fumigation with EOs released from hydrogel formulation. The tested EOs included either (R)-linalool, (S)-linalool, racemic linalool, or a binary mixture of (R)-linalool with one of twelve other essential oils. It was found that the least saturated hydrogels conditioned in essential oils were the most effective, and both species of thrips were more sensitive to (R)-linalool than to (S)-linalool; *F. occidentalis* was significantly more resistant to all treatments than *F. insularis*. The study demonstrates that essential oils in combination with hydrogels are a promising alternative to conventional insecticides for thrips control.

Cockroach pesticide baits—based on locally available natural compounds—have been also explored [135]. The use of EOs on urban pests has been investigated in the USA [94], particularly on cockroaches. Oladipupo et al. [98] tested how hydrogels prolong bioavailability of various essential oil components (limonene, carvacrol, and β-thujaplicin,) to control German cockroach (*Blattella germanica*). The study revealed that limonene, carvacrol, and β-thujaplicin in SAP gels show promising potential to reduce adult male survival/longevity, suppress egg hatchability and female fecundity, and delay the interoothecal period.

### 9.2. Hydrogels Used as Carriers for Natural (EOs) Repellents

Although the repellent effects of essential oils have long been recognized, their use in an unformulated state may not always be suitable. One of the means of prolonging essential oils’ efficacy and making them easy to apply is their incorporation into a polymer or gel matrix. Historically, most research on the application of incorporated essential oils in gelatinous (alginate) matrices, often in combination with the oil encapsulation, has been aimed at mosquito control [136,137] and some other human ectoparasites [138]. These formulations are suitable for fabric or textile impregnation, and they proved to have high wash durability [139]. Oydele et al. [140] prepared ointment and cream formulations of lemongrass oil as mosquito repellent. Apart from mosquitos, there are few research or practical uses of gel formulations with essential oils against stored product pests. Abd-El-Bar and Fawki [141] used gelatin capsules as carriers of three essential oils and tested their insecticidal activity against the storage beetle pest *Acanthoscelides obtectus*.

A special case of the use of essential oils is their incorporation into a glue or adhesive for food packaging production [142]. Before stiffening, the glue is viscous, and after packaging is finished, the glue poses a repellent effect against stored product pests. Repellent systems based on essential oils in gel matrices are also promising in sustainable agriculture [143]. Picard et al. [144] incorporated oils of *Thymus vulgaris* and *Satureja montana* into polymer alginate matrices and found that the polymer repelled western flower thrips, *F. occidentalis*, for 4 days. Recently, de Oliveira et al. [134] used hydrogel-based repellent systems containing botanical compounds against two agricultural pests, the whitefly *Bemisia tabaci* and the spider mite *Tetranychus urticae*.

As already mentioned above, essential oils as repellents were also suggested for the risk management of rodents [67].

## 10. Outlooks and Perspectives

The pesticide industry is currently facing several major challenges. Among the most important challenges are loss of active ingredients due to consumer perception and changing society needs and increasing pest resistance to multiple active pesticide ingredients. The decreasing number of active substances is to some extent compensated by the development of new pesticide formulations with increased bioactivity or more efficient transport of the active substance to the sites of action. New or innovative gel formulations may be one of the options—in addition to traditional pesticide formulations—to address the above problems and challenges. For example, the presented review shows that various gel and hydrogel carriers of insecticides as well as biological control agents (micro-organisms) have already been proposed and constructed for control of several pest species.

**Figure 2:**
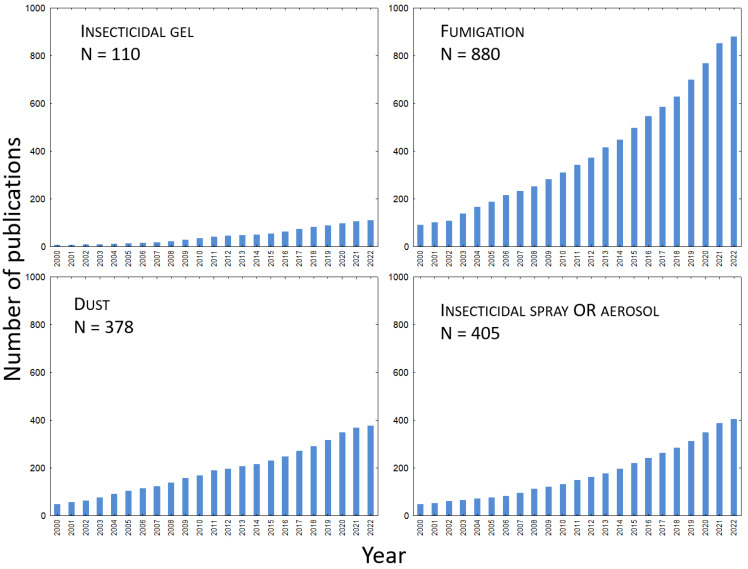
Comparison of cumulative number of WoS peer-reviewed scientific publications from 2000 to the present on the topic of gels and other pesticide formulations (solid dusts, gas-fumigants, liquid sprays) regarding their use for pest control.

What can be expected of the use of gel formulations in the near future? One possible criterion that can help to estimate the future trend of development of gel pesticide formulations is a bibliometric analysis comparing pesticide gels with other formulations. Figure 2 compares publications excerpted from the WoS database regarding gel vs. other types of pesticide formulations such as solid, gas, liquid pesticide formulations. This figure clearly shows that research on non-gel formulations is orders of magnitude more numerous. This indicates that research and practical applications of gels as pesticides form—although they play a key role in certain areas—only a narrow niche and thus still await their wider development and finalization into practical applications. In our opinion, the exploitation of this potential can be expected in the areas mentioned in the following paragraphs.

Without much speculation and large predictive uncertainties, further development of pesticide gel formulations can be expected in those areas in which they are already in use and have proven themselves. This is particularly the case in the areas of the design of innovative or new types of gel insecticide and soft rodenticide baits or mosquito repellent formulations. Innovative controlled-release gel formulations of traditional insecticides, such as carbamates [5] or organophosphates [6], have also been designed for the direct control of field agricultural pests. However, it is questionable what application of these types of gel formulations will find practical use due to increasingly stringent registration measures against these and possibly other groups of neurotoxic pesticide compounds. Thus, a more optimistic outlook for agricultural pest control seems to be in the development of gel formulations as carriers of microbial bio-agents [47], natural botanical insecticides (plant extracts, essentials oils) [46], natural repellents, and gel-based lures for the controlled release [132] of pheromones applied as pheromone mating disruptors [37,145,146] within the framework of IPM strategy. Gels and other polymers (especially biopolymers) then also have good prospects as carriers of environmentally acceptable impregnating agents (e.g., borates) for structural/construction wood protection [111,147,148,149].

Currently, in urban insects, gel baits are generally the preferred pesticide formulations, compared to insecticide sprays and dusts, due to their instrumentally undemanding application methods, potential for secondary transmission, low acute toxicity, minimal nontarget effects, and low environmental contamination [150]. The most intensive development can be expected in the field of insecticidal baits, which will include hydrogels as carriers. [4]. Combinations of gel insecticide baits with controlled release of pheromones seems to also be promising approach. For example, it has been shown for ant control that the addition of a synthetic trail pheromone ((Z)-9-hexadecenal) to hydrogel baits could further increase bait efficacy [151] before the hydrogels lose too much moisture [152]. In addition, species-specific pheromones can also further improve target specificity of the bait.

Of the new developments in rodent control [153], the most obvious trend is that rodenticide manufacturers and users are increasingly favoring the development and use of soft rodenticide baits [22]. This is due to their ease of application, attractiveness, and palatability. Soft types of baits may gain further market space, as the registration of many types of drinkable anticoagulant baits has not been extended in the EU and other countries. Most importantly, the new generation of soft gel and paste bait formulations may reduce the risks of uncontrolled bait transport by rodents from bait stations and their uncontrolled environmental contamination. Gels and pastes have potential as they represent suitable formulations from a food industry perspective since they can be specifically constructed for allergen-free operations. In addition, from the available scientific documentation, it appears that soft rodenticide baits are less susceptible to infestation and spoilage by storage arthropods [154]. Species-specific RNAi-based agents, for which gels and pastes can be used as carriers, are considered a future alternative to toxic baits [155].

Currently, arthropod resistance to insecticides is becoming a serious problem worldwide [156,157,158,159]. In the field of pest management, molecular methods are mainly used for the diagnosis of harmful organisms [160], while as methods for direct pest control these new approaches—with the exception of GMOs—have only a limited sphere of application. However, resistance to traditional active ingredients may provide an opportunity to develop molecular methods and formulations to control pests, including various forms of nanoformulations [161] and polymers and the use of gels. Although the results are still in the research stage, they do not look pessimistic. For example, a substantially new generation of bait technologies for liquid baits with liposomes as carriers of dsRNA has been suggested by several research teams [95,96]. Specifically, a new method was developed [95] which uses liposome vesicles (so-called dsRNA lipoplexes) as carriers of dsRNA molecules. Laboratory tests showed that the protected molecules (lipoplexes), formulated as liquid or gel baits, may trigger lethal RNA interference in the cockroach digestive system. Oral-delivery-mediated RNAi was first used to silence the LeVgR gene in *Liposcelis entomophila* (Enderlein) [96]. The VgR gene may thus become an important potential target for disrupting insect reproduction for pest management through the oral delivery of dsRNA. Owing to these new approaches, there is a real chance that in the future, such types of technologies could become the basis for “smart” pest control products delivered via gel baits.

## 11. Conclusions

Various gel carriers of rodenticides and insecticides, as well as biological control agents (microorganisms), have been proposed for the control of many pest species. Traditional granular formulations for the control and/or monitoring of rodents have recently started to be replaced by paste and gel formulations. Various gel bait formulations have been successfully used for several decades to control public health and urban pests, such as cockroaches and ants; new hydrogel-based formulations are mainly in the research and development stage. Profound advances have been made in the use of gel formulations as repellents in the management of the medical risks of mosquitoes as vectors of pathogens such as malaria. Despite research effort, few if any practical formulations of traditional neurotoxic insecticides seem to be available and registered for the control of agricultural and forest pests. However, gel nanoformulations were constructed for the slow release of pheromones for behavioral manipulation of horticultural pests. The potential of gels for application of plant extracts (e.g., essential oils, EOs) in agriculture is being newly explored; hydrogels were suggested as an emerging technology for controlled fumigation release of EOs for plant pest control in greenhouses, e.g., resistant plant pests. Promising results have been obtained for the protection of foodstuffs such as dried ham by harmful mites using gel formulations. Current research shows that gel formulations would find significant application as carriers of so-called “smart insecticides”, which are based on molecular disruptors of iRNA.

## Data Availability

Not applicable.
